# Outcomes of Pediatric Patients with Crohn's Disease Received Infliximab or Exclusive Enteral Nutrition during Induction Remission

**DOI:** 10.1155/2022/3813915

**Published:** 2022-09-02

**Authors:** Yao Lv, Yue Lou, Gan Yang, Youyou Luo, Jingan Lou, Qi Cheng, Jindan Yu, Youhong Fang, Hong Zhao, Kerong Peng, Jie Chen

**Affiliations:** Gastroenterology Department, Children's Hospital, Zhejiang University School of Medicine, National Clinical Research Center for Child Health, Hangzhou, China

## Abstract

**Background:**

Both exclusive enteral nutrition (EEN) and infliximab (IFX) are recommended as induction therapy for pediatric Crohn's disease (CD). Our aim was to compare long-term disease outcomes of patients initially received with either IFX or EEN.

**Methods:**

Medical records of newly diagnosed, therapy naïve pediatric patients with CD received with IFX or EEN as induction therapy were retrospectively enrolled. Pediatric Crohn's disease activity index (PCDAI), Crohn's disease endoscopic index of severity (CDEIS), and other clinical data were compared pre- and postinduction therapy in two groups. The sustained remission rates and time coupled with body mass index (BMI) and height for age (HFA) changes were evaluated during more than 2-year long-term follow-up.

**Results:**

We collected data from 58 children with CD used IFX (23) or EEN (35) as induction remission therapy from January 2015 through June 2021 in our single-center. The median follow-up after starting IFX or EEN was 12.2 months (6.5–18.0months) and 18.9 months (7.1–30.7months), respectively. The proportion clinical and endoscopic remission in EEN (88.57% and 68.75%) was similar with that of IFX (73.91% and 80.77%) after induction therapy. No significant differences were also observed in BMI and HFA recovery between two groups. Among those who achieved clinical or endoscopic remission or endoscopic response, the sustained remission rates and time did not reveal any significant differences for those 10 patients who used 6-mercaptopurine/methotrexate (6-MP/MTX) or 14 patients who used IFX as maintenance treatment during longitudinal follow-up.

**Conclusions:**

Our study suggested that EEN treatment is similar with IFX therapy in short-term outcomes, and EEN+6-MP/MTX treatment is comparable with IFX+IFX therapy in long-term outcomes.

## 1. Introduction

Crohn's disease (CD) is relapsing systemic inflammatory disease, affects the whole gastrointestinal tract, and may cause extraintestinal complications associated with immune disorders [1, 2]. Pediatric CD has increased in both incidence and prevalence all over the world in the past two decades [3]. The precise etiology for CD is still unknown, and no complete cure possibilities for it have been discovered so far [4]. Moreover, pediatric-onset patients usually have risk for faltering growth [5]. Therefore, optimal treatment strategies are always desired with hope to relieve symptoms, to promote mucosal healing (MH), to optimize growth, to improve quality of life, and avoid or reduce recurrence with trivial side effects.

The current guidelines for the induction therapy in pediatric CD mainly include EEN, corticosteroids, anti-TNF therapy [6]. EEN is recommended as the first-line induction remission therapy for mild to moderate pediatric CD, offering a complete liquid diet consists of polymeric or elemental formula, able to induce remission in up to 89% of patients with CD [6]. EEN is clinically efficacious and associated with improvements on mucosal healing, linear growth, and bone health in the period of induction remission treatment [7–9]. However, full enteral feeding is not well tolerated, resulting in the failure of the therapy frequently in practice, and the long-term effectiveness of EEN remains uncertain.

MTX, thiopurines (azathioprine or 6-MP), maintenance enteral nutrition, anti-TNF agents are suggested as maintenance therapy for pediatric CD [6]. MTX is the first-choice immunomodulator in maintaining clinical remission, and its pooled maintenance clinical remission rate is 37.1% [10], but it can cause nausea and vomiting during maintenance use [6]. If MTX is ineffective or intolerant, then 6-MP can be used to maintain remission for those reach remission; however, haematological toxicity and pancreatitis are major problems at start of therapy [11]. IFX, an antitumor necrosis factor alpha (TNF-*α*) agent, is employed for both induction and maintenance of remission therapy among the patients with moderate to severe CD [12]. It was reported that IFX was superior to conventional treatment in achieving short-term clinical and endoscopic remission [13]. Despite these benefits, the formation of antidrug antibodies after repeated administration and loss of response may lead to treatment failure [14]. Moreover, acute infusion reactions (AIR), opportunistic infections, and risk of malignancy are the main concerns of the patients who are receiving IFX [15]. The long-term side-effects of this agent have not yet been fully investigated.

It is reported that EEN was as effective as corticosteroids in induction remission [16]. A randomized controlled trial (RCT) in pediatric patients with moderate-to-severe CD suggested more IFX-treated patients than EEN or prednisolone-treated patients were in clinical remission and endoscopic remission at week 10. However, the proportion of patients in clinical remission was similar at week 52 between the two groups [13]. In maintenance remission, there were no differences between patients initially treated with EEN or corticosteroids [17].

Here we reported real-life experience and compared the short-term efficacy of EEN and IFX and long-term efficacy of EEN+6-MP/MTX therapy and IFX+IFX therapy in pediatric CD. Our aim was to determine which agent is superior as induction remission therapy for mild to moderate pediatric CD with long-term clinical outcomes.

## 2. Materials and Methods

### 2.1. Patient Enrollment

This was a retrospective cohort study enrolled 58 patients conducted at the Children's Hospital, Zhejiang University School of Medicine, a major referral center for children with inflammatory bowel disease (IBD) in China from January 2015 to June 2021. Enrolled patients were under the age of 18 years, newly diagnosed, therapy naïve CD on the basis of ESPGHAN Revised Porto Criteria [18] according to clinical manifestations, endoscopic appearance, biopsies, and radiological findings used EEN for 2 months or IFX for 3 times as induction of remission therapy. The exclusion criteria for short-term comparison were defined as follows: (1) children who had not continuously received EEN therapy or infliximab treatment for more than 2 months or 3 times, respectively, (2) patients who could not tolerate adequate doses of IFX, (3) subjects failed to achieve daily amount of formula for more than 3 days, and (4) patients with PCDAI ≥ 40 points. The following were included for long-term comparison criteria: (1) patients who achieved PCDAI < 10 or CDEIS < 3 or a decrease in CDEIS score more than 5 points at the end of induction remission therapy, (2) more than once follow-up was required within one year or during longitudinal follow-up, and (3) IFX group and EEN group solely received IFX or 6-MP/MTX as maintenance therapy.

### 2.2. Study Design

Included patients were divided into two treatment groups. The IFX group received three intravenous IFX (Inflectra, CT-P13) infusions of 5-10 mg/kg induction therapy at weeks 0, 2, and 6 and followed by maintenance treatment. The EEN group used polymeric feeding as induction treatment for 2 months followed by maintenance treatment. Both IFX group and EEN group used IFX or 6-MP (1.0~1.5 mg/(kg/d))/MTX (10~25 mg/m^2^/week) as maintenance therapy. The choice of induction therapy and maintenance treatment was based on patient preference, in accordance with the physicians. The disease activity and the endoscopic response were measured by the PCDAI [19] and Crohn's disease endoscopic index of severity CDEIS [20], respectively. Disease distribution and phenotypes were classified by the Paris criteria [21]. Mucosal healing was evaluated through the CDEIS: complete endoscopic remission (CDEIS score less than 3) and endoscopic response (a decrease in CDEIS score more than 5 points) [22]. The activity of disease was reflected by PCDAI: PCDAI < 10: remission, PCDAI 10–27.5: mild disease, PCDAI 30–37.5: moderate disease, and PCDAI 40-100: severe disease [23]. When compared the recurrence time by Kaplan–Meier curve analysis, the patients did not achieve PCDAI score or CDEIS score more than 10 or 3, respectively, during maintenance therapy. Data for all patients were derived from their inpatient electronic medical records. The study was approved by the Medical Ethical Committee of Children's Hospital, Zhejiang University School of Medicine under 2019-IRB-109 of 27 August 2019. The children and their legal guardians both signed the consent for the use of their information in this study in accordance with this approval. The study complies with the ethical guidelines of the 1975 Declaration of Helsinki as reflected in the prior approval of the Agency's Human Research Committee.

## 3. Statistical Analysis

Statistical analysis was performed using IBM SPSS 17.0 (SPSS Inc., USA). Data were presented as mean ± standard deviation (SD). Comparison of parametric continuous variables was performed by using the Mann-Whitney *U* test or Student's *t*-test, as appropriate. The chi-squared or Fisher's exact test was used to compare categorical variables. Kaplan–Meier survival curves were used to compare recurrence time between EEN+6-MP/MTX and IFX+IFX groups, and the results were present as hazard ratios (HR) with 95% confidence intervals (CI). *P* value of < 0.05 was considered to be statistically significant. *P* value of < 0.01 was considered highly significant.

## 4. Results

### 4.1. Baseline Characteristics per Treatment Group

The demographical and clinical features of the 58 patients with 23 initiating IFX and 35 EEN included in the research are shown in Table 1. The age, height, weight, BMI *z*-score, HFA *z*-score, PCDAI score, CDEIS score, disease location, disease behavior, disease growth, laboratory information including erythrocyte sedimentation rate (ESR), C-creative protein (CRP), and albumin (Alb) did not differ significantly between the patients treated with IFX or EEN at the baseline. Most patients in the IFX group were females but the gender distribution of the EEN group was almost in each half. The rates of perianal disease were higher in patients receiving IFX compared with the patients receiving EEN (*P* < 0.01).

### 4.2. Clinical Response and Remission after Induction Therapy in Two Groups

**Figure 1:**
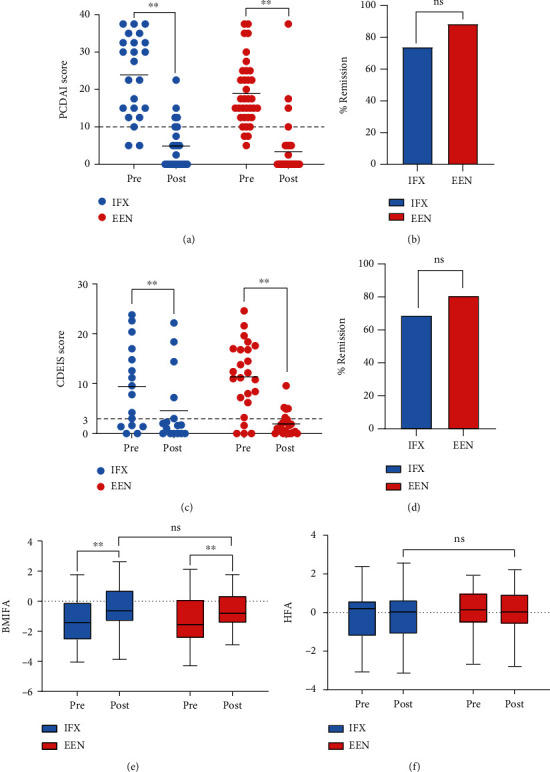
Comparisons of short-term disease outcomes among the two groups after induction remission therapy. (a) PCDAI scores and (c) CDIES scores of IFX- or EEN-treated patients at pre and postinduction therapy. Paired Student's *t*-test. Percentage of (b) IFX- or (d) EEN-treated patients in clinical remission (PCDAI < 10) and endoscopic remission (CDEIS < 3) after induction remission strategies. (e) BMIFA and (f) HFA of patients treated with EEN or IFX at diagnosis and at the end of induction therapy. Chi-squared or Fisher's exact test. ^∗^*P* < 0.05; ^∗∗^*P* < 0.01.

The PCDAI score, CDEIS score, BMI *z*-score, HFA *z*-score, and other laboratory values such as ESR and CRP were used to compare the efficacy of induction therapy among the groups. From our data, there was no statistical difference in clinical remission defined as PCDAI < 10 (IFX 73.9% (17/23) vs. EEN 88.6% (31/35), *P* = 0.153) at the end of induction therapy (Figure 1(b)), although both two groups achieved an obvious decrease in PCDAI score at postinduction when compared with that of preinduction (Figure 1(a)). There was no statistically significant pairwise difference in inducing mucosal healing evaluated by the CDEIS < 3 between EEN groups (80.8% and 21/26) and IFX groups (73.3% and 11/16), *P* = 0.374 (Figure 1(d)). Both treatment groups saw a decrease in CDEIS after primary induction therapy (Figure 1(c)). Nutritional status and growth recovery evaluated by changes in BMI and HFA were similar in two groups at the end of induction therapy (Figures 1(e) and 1(f)).

### 4.3. Clinical Features of Enrolled Patients at the Beginning of Maintenance Therapy Period

**Figure 2:**
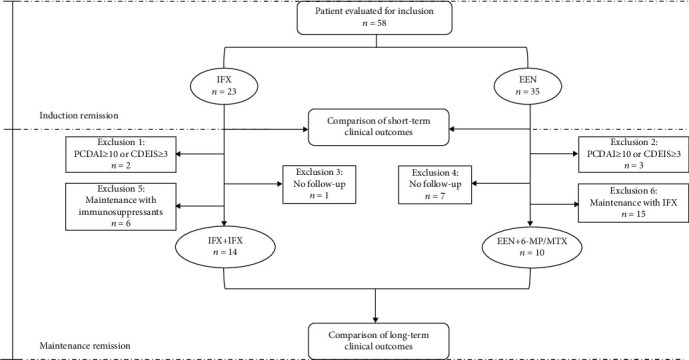
Study outline and patient selection. IFX+IFX: IFX was used as monotherapy during both the period of induction and maintenance; EEN+6-MP/MTX: EEN was used as induction therapy, and 6-MP/MTX was used as monotherapy during maintenance therapy.

After induction remission therapy, 10 of 35 children in the EEN group and 14 of 23 patients in the IFX group reached the long-term comparison standard (Figure 2). Among the 24 patients, the PCDAI, CDEIS, BMIFA, HFA, gender distribution, disease location, and behavior were similar between the two groups at the beginning of induction remission therapy (Table 2). As shown in Table 2, perianal disease occurred in 10/14 (71.4%) IFX+IFX group and 0/10 (0%) EEN+6-MP/MTX group (*P* < 0.01). All the patients in the IFX+IFX group used IFX in the period of maintenance therapy, while on the contrary, all patients in the EEN+6-MP/MTX group received 6-MP or MTX as maintenance therapy (*P* < 0.01). None of the children received partial enteral nutrition (PEN) during maintenance therapy.

### 4.4. Comparison of Sustained Remission Rates and Time among the Two Groups

**Figure 3:**
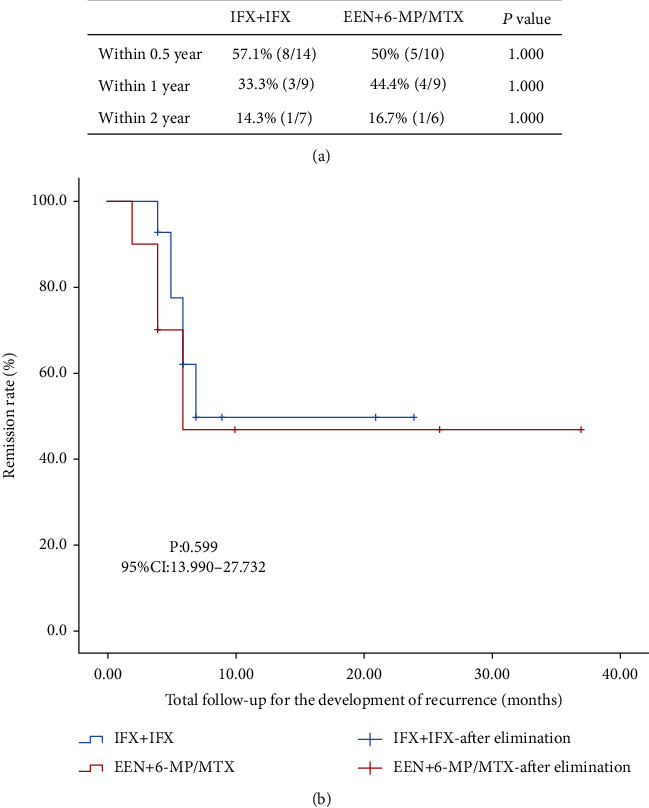
Comparison of sustained remission effect in each treatment group. (a) Sustained remission rate at 0.5, 1, and 2 years after induction therapy between IFX+IFX and EEN+6-MP/MTX groups. (b) Kaplan–Meier curve showing sustained remission time from maintenance treatment to first recurrence. The log-rank test was used to compare differences among the groups.

There was no significant difference in sustained remission rates estimated by PCDAI < 10 and CDEIS < 3 in IFX+IFX and EEN+6-MP/MTX groups during the first 0.5, 1, and 2 years after primary induction (Figure 3(a)). To further evaluate sustained remission time of the two groups. Kaplan–Meier curve was used to compare the clinical or endoscopic recurrence time after the induction therapy (Figure 3(b)). No significant difference between the two groups was observed during longitudinal follow-up (*P* = 0.599, 95% CI: 13.990–27.732).

### 4.5. Comparison of Linear Growth and Nutritional Status between the Two Groups during More than 2-Year Follow-Up

**Figure 4:**
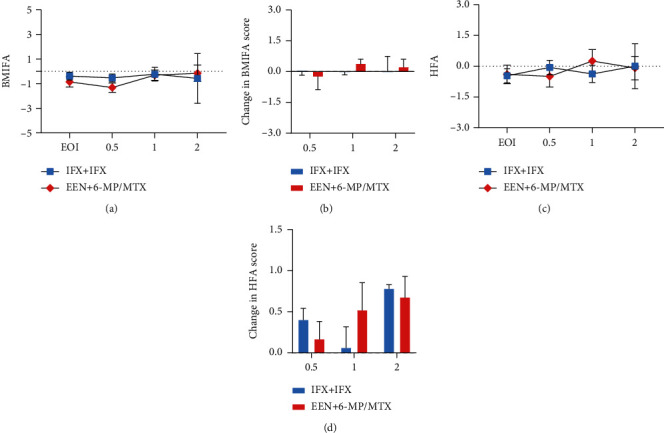
The linear growth outcomes and nutritional status during longitudinal follow-up. (a) BMI *z*-score and (b) HFA *z*-score of children treated with EEN+6-MP/MTX or IFX+IFX and 0.5-, 1-, and 2-year follow-up. Changes in (c) BMI *z*-score (*Δ*BMI) and (d) HFA *z*-score (*Δ*HFA) comparing with the end of induction (EOI) remission therapy during maximum 2-year follow-up. ^∗^*P* < 0.05; ^∗∗^*P* < 0.01.

Both the nutritional status estimated by BMIFA and change in BMIFA remained comparable between IFX+IFX and EEN+6-MP/MTX groups during the first 0.5, 1, and 2 years after primary induction (Figures 4(a) and 4(b)). No obvious increase in linear growth and no changes in HFA of the CD patients receiving 6-MP/MTX or IFX for maintenance therapy were observed over the follow-up period (Figures 4(c) and 4(d)), although it seems that the EEN+6-MP/MTX group showed a positive (>0) change in BMIFA at 1- and 2-year follow-up after the induction therapy (Figure 4(b)). On the contrary, both two groups saw a positive (>0) change in HFA during the follow-up period (Figure 4(d)).

## 5. Discussion

This is the first pediatric cohort study evaluating short-term outcomes of IFX and EEN induction therapy and long-term outcomes of EEN+6-MP/MTX and IFX+IFX treatment. Our study demonstrates that EEN was similar with IFX in induction therapy period for mild-to-moderate pediatric CD patients, and the long-term disease outcomes of EEN+6-MP/MTX and IFX+IFX including sustained remission rates and time, linear growth, and nutritional status were also comparable.

From our data, clinical and endoscopic remission rates after EEN or IFX induction remission therapy were not statistically different for mild-to-moderate pediatric patients with CD in our study. These results were consistent with our previous small sample research [24]. Consistent with other studies [25, 26], both EEN and IFX groups saw a dramatic improvement of nutritional status and a decrease in inflammatory status (Fig s1) during induction therapy period, although more perianal disease and less female children at diagnosis were in those treated with IFX. Based on the ECCO-ESPGHAN guidelines for the medical management of pediatric Crohn's disease [6], EEN is recommended for patients with low-risk luminal CD whereas IFX is recommended for patients with perianal disease, penetrating behavior or severe growth retardation. The difference on the choice of EEN or IFX cannot be avoided in the real-life events; in our data, 3 children with perianal disease were still treated with EEN, which was because these patients preferred to receive EEN therapy in consideration of the price or the risk of infections and malignancy caused by biologic agents. In the pediatric population, long-term disease outcomes such as modification of disease progression especially guarantee of suitable growth and development must be taken into account before short-term therapy to achieve remission be carried out. However, rare previous research has compared the long-term outcomes of patients initially treated with EEN and IFX. From our data, patients initially treated with IFX and EEN had a similar clinical remission on long-term outcomes, which was consistent with other results [13]. From our data, 10 of 35 patients in the EEN group received an immunomodulator (6-MP/MTX) and 14 of 23 children from the IFX group used biological agents (IFX) during maintenance therapy (Figure 2). Our analysis indicated at 0.5 year endpoint, sustained remission rates favoured IFX+IFX versus EEN+6-MP/MTX treatment (57.1 vs. 50%) but not were statistically different (Figure 3(a)). Both groups saw a down trending sustained remission rates as time goes by with no significant difference. In keeping with the comparable rates of maintenance of remission, there was no significant effect of the therapy (IFX+IFX vs. EEN+6-MP/MTX) on achieving sustained remission time (Figure 3(b)). Impaired growth is common in patients with CD, specific to the pediatric population [27]. Within our study cohort, patients treated with IFX did not exhibit significantly greater improvement in BMIFA and HFA than 6-MP/MTX-treated patients by 0.5-, 1-, and 2-year follow-up (Figure 4). In conclusion, patients treated with IFX had no benefit of growth improvement. The IFX+IFX group presented with high percent of perianal disease relative to the EEN group at the beginning of maintenance treatment period. Thus, our results further support that EEN plus traditional immunomodulator (6-MP/MTX) is feasible for mild to moderate pediatric CD without increased need for escalation to anti-TNF therapy.

The current study is limited by the retrospective design. Therefore, better matched, prospective, and larger number of multicenter cohort study was required in the future to control for baseline demographic characteristics.

In conclusion, despite limitations related to the retrospective study design, the current study demonstrates that for mild to moderate pediatric CD patients, the use of EEN leads to comparable clinical and endoscopic rates of remission, BMI, and HFA recovery compared with IFX during initial induction therapy. As for long-term disease outcome, our data do not suggest that patients will benefit from biological agent IFX versus traditional immunomodulator 6-MP or MTX.

## Figures and Tables

**Table 1:** Demographic and clinical data at baseline of per treatment group.

	IFX	EEN	*P* value
*n*	23	35	
Age (yr)	12.73 ± 1.93 (7.33-15.25)	12.16 ± 1.84 (7.67-15.58)	0.16
Males/females	21/2	22/13	0.01
Height (cm)	153.40 ± 11.74	150.87 ± 14.61	0.50
Weight (kg)	38.64 ± 7.14	37.85 ± 12.70	0.77
HFA *z*-score	−0.22 ± 1.34	−0.03 ± 1.18	0.43
BMIFA *z*-score	−1.25 ± 1.67	−1.26 ± 1.68	0.92
PCDAI score	23.91 ± 10.87	18.93 ± 8.65	0.09
CDEIS score	7.87 ± 7.86	8.73 ± 7.37	0.70
Disease location, *n*(%)			0.43
L1	8	8.2	
L2	0	1.4	
L3	32	41.1	
L4a	26	13.7	
L4b	34	35.6	
Disease behavior, *n*(%)			0.24
B1	84.21	94.29	
B2	15.79	5.71	
Perianal disease, *n*(%)	56.52	8.57	<0.01
Disease growth, *n*(%)			0.85
G0	86.96	88.57	
G1	13.04	11.43	
Alb (g/L)	36.09 ± 5.13	37.18 ± 5.19	0.43
ESR (mm/h)	39.48 ± 25.88	42.00 ± 27.62	0.66
CRP (mg/L), normal (%)	43.48	22.86	0.10

IFX: infliximab; EEN: exclusive enteral nutrition; PCDAI: pediatric Crohn's disease activity index; CDEIS: Crohn's disease endoscopic index of severity; BMIFA: body mass index for age; HFA: height for age; Alb: albumin; ESR: erythrocyte sedimentation rate; CRP: C reactive protein.

**Table 2:** Demographic and clinical characteristics of enrolled patients at the beginning of maintenance therapy.

	IFX+IFX	EEN+6-MP/MTX	*P* value
*n*	14	10	
Age (yr)	13.61 ± 1.22 (11.17-15.58)	12.71 ± 2.18 (8.75-15.75)	0.32
Males/females	13/1	8/2	0.55
Height (cm)	156.67 ± 8.53	152.85 ± 14.07	0.42
Weight (kg)	44.63 ± 7.26	40.15 ± 10.08	0.22
HFA *z*-score	−0.47 ± 1.28	−0.13 ± 1.22	0.56
BMIFA *z*-score	−0.38 ± 1.15	−1.00 ± 1.22	0.24
PCDAI score	3.21 ± 3.85	1.75 ± 2.37	0.40
CDEIS score	0.65 ± 0.82	2.00 ± 3.47	0.44
Disease location, *n*(%)			0.21
L1	9.4	6.3	
L2	0	0	
L3	28.1	50	
L4a	28.1	6.3	
L4b	34.4	37.5	
Disease behavior, *n*(%)			0.48
B1	81.8	100	
B2	18.2	0	
Perianal disease, *n*(%)	71.4	0	0.00
Disease growth, *n*(%)			1.00
G0	85.7	80	
G1	14.3	20	
Alb (g/L)	44.33 ± 9.40	44.68 ± 1.91	0.91
ESR (mm/h)	9.29 ± 10.84	12.33 ± 6.95	0.12
CRP (mg/L), normal (%)	100	100	
Maintenance therapy (%)			0.00
IFX	100	0	
6-MP/MTX	0	100	
Used PEN during the maintenance therapy	No	No	>0.05

IFX: infliximab; EEN: exclusive enteral nutrition; PCDAI: pediatric Crohn's disease activity index; CDEIS: Crohn's disease endoscopic index of severity; BMIFA: body mass index for age; HFA: height for age; Alb: albumin; ESR: erythrocyte sedimentation rate; CRP: C reactive protein; 6-MP: 6-mercaptopurine; MTX: methotrexate; PEN: partial enteral nutrition.

## Data Availability

Individual participant data will not be shared.

## Supplementary Materials

Supplementary Materials Supplementary figure: the laboratory data ESR at baseline (BSL) and postinduction treatment. Paired Student's *t*-test. ^∗^*P* < 0.05; ^∗∗^*P* < 0.01.
