# Supplementary figures and images for: Outcomes of Pediatric Patients with Crohn's Disease Received Infliximab or Exclusive Enteral Nutrition during Induction Remission
Source: Gastroenterol Res Pract. 2022 Sep 2;2022:3813915. doi: 10.1155/2022/3813915 (PMC9462978; doi:10.1155/2022/3813915)

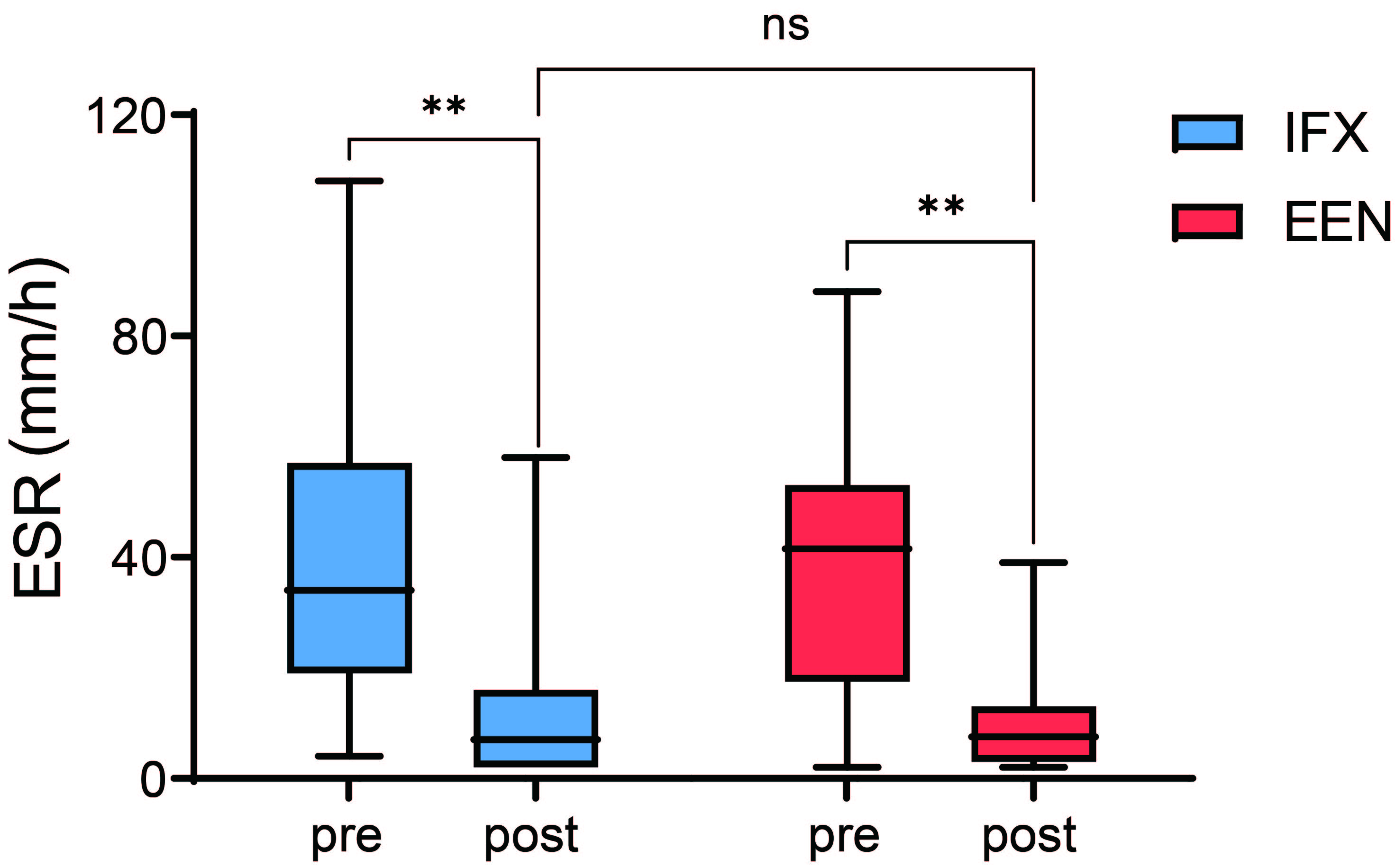

Supplement: Supplementary Materials — Supplementary figure: the laboratory data ESR at baseline (BSL) and postinduction treatment. Paired Student's t-test. ∗P < 0.05; ∗∗P < 0.01. [file 3813915.f1.jpg]
